# Active Joint Mechanism Driven by Multiple Actuators Made of Flexible Bags: A Proposal of Dual Structural Actuator

**DOI:** 10.1155/2013/128916

**Published:** 2013-12-09

**Authors:** Hitoshi Kimura, Takuya Matsuzaki, Mokutaro Kataoka, Norio Inou

**Affiliations:** Department of Mechanical and Control Engineering, Tokyo Institute of Technology, Ookayama 2-12-1, Meguro-ku, Tokyo 1528552, Japan

## Abstract

An actuator is required to change its speed and force depending on the situation. Using multiple actuators for one driving axis is one of the possible solutions; however, there is an associated problem of output power matching. This study proposes a new active joint mechanism using multiple actuators. Because the actuator is made of a flexible bag, it does not interfere with other actuators when it is depressurized. The proposed joint achieved coordinated motion of multiple actuators. This report also discusses a new actuator which has dual cylindrical structure. The cylinders are composed of flexible bags with different diameters. The joint torque is estimated based on the following factors: empirical formula for the flexible actuator torque, geometric relationship between the joint and the actuator, and the principle of virtual work. The prototype joint mechanism achieves coordinated motion of multiple actuators for one axis. With this motion, small inner actuator contributes high speed motion, whereas large outer actuator generates high torque. The performance of the prototype joint is examined by speed and torque measurements. The joint showed about 30% efficiency at 2.0 Nm load torque under 0.15 MPa air input.

## 1. Introduction

In a mechanical operation, the prior performance changes depending on the situation. For example, high speed is required to approach an object [1, 2]. In contrast, to convey a heavy object, strong force is more important than the speed of the motion [3]. However, in general, actuator performance is limited by its size and weight. If an actuator satisfies both high-speed and high-strength requirements, it will be enormously large and heavy. In practice, there are mainly two methods to satisfy the speed versus strength trade-off with a single mechanism. One involves a quick change in the reduction ratio, and the other involves a significant change in the actuator output. In terms of the reduction ratio, quick and steady motion is not easy although there are many studies on reduction mechanisms such as continuously variable transmission (CVT) [4, 5]. In addition, complexity of the mechanism and weight increase are also the problems associated with this method. Achieving a significant power change is also difficult because actuator performance is limited in practice. Configuring multiple actuators for one driving axis seems to be an effective method; however, it is almost impossible to use multiple motors for one axis because of the output matching problem. To solve this problem, Kim et al. propose a multiactuator mechanism with a planetary gear [6, 7]. Hu et al. also propose a similar mechanism using a differential gear [8]. Ma et al. propose using coupled actuators for the *n* DOF joint driven by *n* actuators to achieve high power and efficiency [9]. These mechanisms are types of variable power mechanisms; however, the mechanical arrangement is constrained. In addition, free rotation is difficult because of the reduction mechanism of gearhead motors.

**Figure 1:**
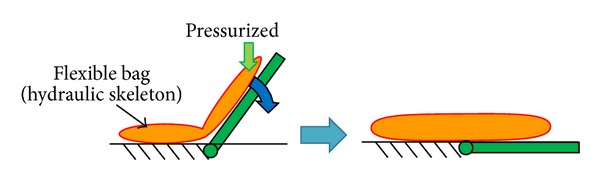
Basic principle of hydraulic skeleton actuator.

This study proposes a new multiple actuator mechanism with a hydraulic skeleton. Hydraulic skeletons are found in living things such as earthworm and actinia. The hydraulic skeleton actuator in this study is made of flexible bags. Compared with former studies on flexible actuators [10–13], the objective of this study is coordinated motion of multiple actuators by using flexibility for output matching. The driving force of the actuator is generated as the bending torque of the bag with this mechanism, as shown in Figure 1. With the proposed mechanism, the torque of the actuator is controlled by the inner pressure.

**Figure 2:**
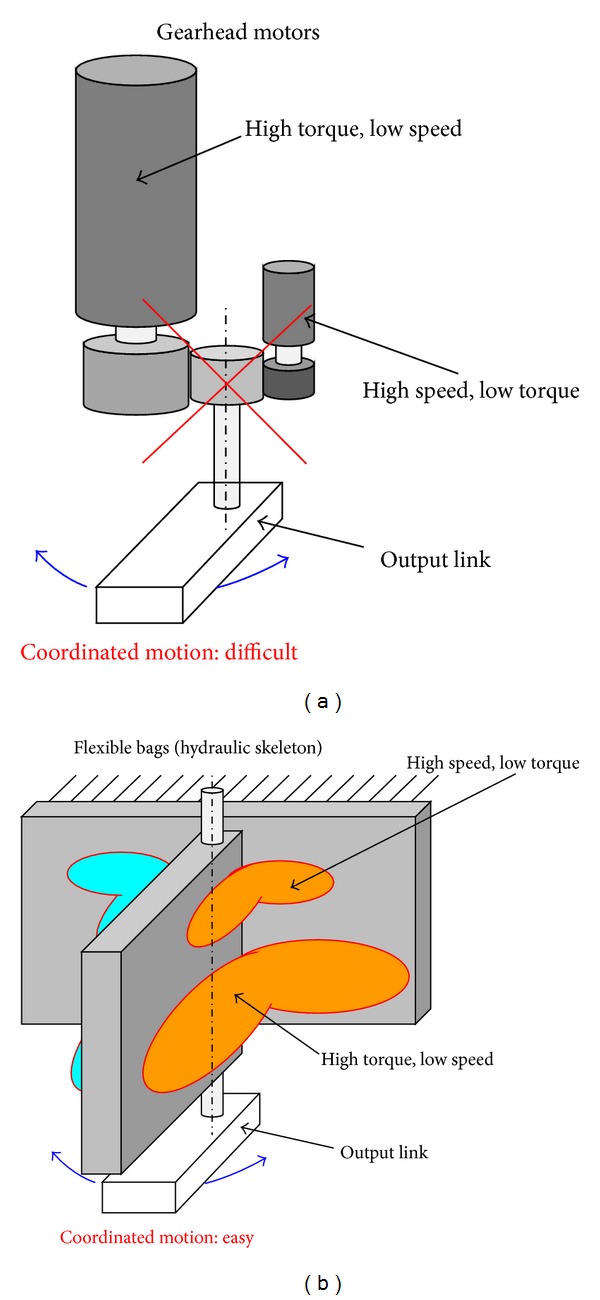
Comparison between conventional and proposed mechanisms.

Figure 2 shows the difference between the conventional mechanism and the proposed mechanism of the multiple actuators. Using gearhead motors with a high reduction ratio is the conventional method of obtaining high torque, for several reasons [14, 15]. However, the output axis becomes stiff when the reduction ratio is high. This stiffness causes the problems of shock resistance and output matching. Moreover, the stiffness of the output axes is different from each other. These are the reasons why using two or more motors for one axis is difficult. On the other hand, the proposed mechanism uses flexible actuators. With this actuator, the stiffness is variable depending on the inner pressure. Thus, output matching could be accomplished very easily because nonactive actuators are flexible when they are depressurized.

Even a high-power actuator is flexible enough if depressurized, and it does not interfere with the other actuator motions. With the proposed mechanism, various arrangements of the actuators are possible compared to the mechanisms with general gearhead motors. The proposed mechanism has further advantages: ease of free rotation, light weight, and good antishock property because of actuator compliance.

This paper discusses a prototype active joint mechanism with a new flexible actuator. The new actuator has dual cylindrical structure. The cylinders are made of flexible bags and have different diameters. The torque of the joint is estimated from the actuator torque and the geometrical relationship between the actuator and the joint. The coordinated motion of the inside and outside actuators is confirmed by a prototype joint mechanism. The torque, the power, and the efficiency are also measured to evaluate the performance of the joint.

## 2. Flexible Actuators and Joint Mechanism

### 2.1. Dual Structural Flexible Actuator

**Figure 3:**
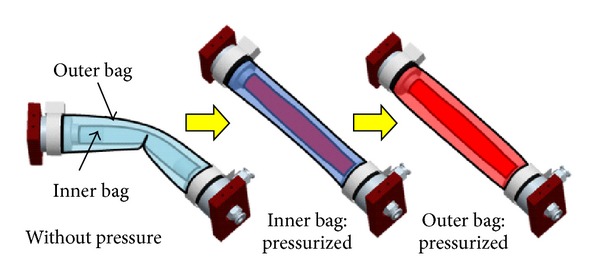
Schematic of dual structural actuator.

The dual structural actuator consists of two cylindrical flexible bags with different diameters. The stiffness of the actuator bag is controlled by the inner pressure. The inner pressure of each bag is controlled independently. The actuator generates the driving force by increasing the inner pressure of the bag, whereas the actuator is flexible under low input pressure.  Figure 3 illustrates the actuator motion. With the dual structural actuator, the inner bag is used for position control with quick motion and the outer bag is required high torque.

The actuator speed depends on the bag size because the maximum flow has an upper limit. The driving force of the actuator has positive relation to the width of the actuator as described in our previous paper [16]. In other words, a small actuator moves fast, whereas a wide actuator generates strong driving force with slow speed in general. The torque of the joint depends on the length between the driving axis and the actuator position because of leverage. With the proposed joint mechanism, the actuator which is fixed near the driving axis is in charge of high speed motion with small torque.

### 2.2. Proposed Joint Mechanism

**Figure 4:**
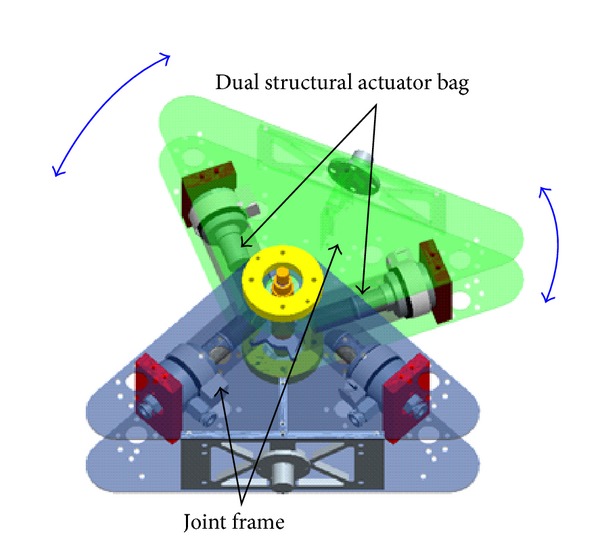
Schematic of the proposed joint mechanism.

The schematic of the proposed joint mechanism is shown in Figure 4. The joint has a couple of the dual actuators to control the joint angle. Both the inner and outer actuators contribute to the rotation of the joint. The inner actuators (hereafter referred to as inner bags) are in charge of position control to approach an object with small torque. In contrast, the outer actuators (hereafter referred to as outer bags) are used for large torque generation after grasping the object. The joint is moved by the coordinated motion of these actuators. The joint angle is maintained by the torque balance of the actuators fixed on both sides. The compliance of the joint can be varied by changing the actuator torque. Free rotation is also possible when all the actuators are depressurized. As described before, the joint has impact resistance because of the actuator flexibility. Further resistance will be achieved by additional countermeasures of the surge pressure of the actuators such as pressure feedback control or the use of a release valve.

## 3. Driving Force Estimation

In our former study, the torque at the bending part of a flexible bag with a rectangular shape was investigated with both nonlinear finite element analysis and experiments [16]. The actuator torque *τ*_*a*_ is approximated by the following equation:

(1) $$\begin{array}{c} \tau_{a}=k_{a}pw^{3}f\left(\theta \right),\;f\left(\theta \right)=\sum_{i=0}^{5}k_{i}\theta^{i}, \end{array}$$

where *k*_*a*_ is a constant of proportionality, *p* is the inner pressure, *w* is the width of the flexible bag, *θ* is the bending angle of the bag, and *f*(*θ*) is empirical formula, which is a polynomial of degree 5, while *k*_*i*_ is constant for each *θ* ^*i*^ [16].

The torque of the dual structural actuator is also estimated from (1). According to this actuator torque, the joint torque can be estimated.

**Figure 5:**
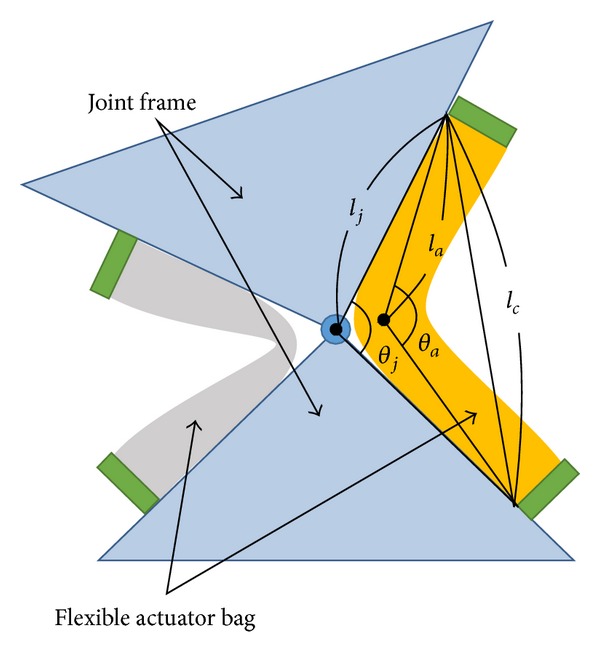
Geometry of the joint and actuator bags.

Figure 5 illustrates the geometry of the proposed joint. The product of the torque and infinitesimal angle change yields the work of the rotational system. The joint and the actuator bag have the same value of the work based on the principle of virtual work:

(2) $$\begin{array}{c} \tau_{j}\Delta \theta_{j}=\tau_{a}\Delta \theta_{a}, \end{array}$$

where *τ*_*j*_ and *τ*_*a*_ are the torque of the joint and the actuator and Δ*θ*_*j*_ and Δ*θ*_*a*_ represent the small variations of their bending angles. This relationship is equivalent to the following formula:

(3) $$\begin{array}{c} \tau_{j}=\tau_{a}\frac{d\theta_{a}}{d\theta_{j}}. \end{array}$$

Here, the relationship between *θ*_*j*_ and *θ*_*a*_ is derived from the law of cosines as shown in Figure 5:

(4) $$\begin{array}{c} l_{j}^{2}\left(1-\cos\theta_{j}\right)=l_{a}^{2}\left(1-\cos\theta_{a}\right)=\frac{l_{c}^{2}}{2}. \end{array}$$

Because the actuator torque *τ*_*a*_ is already approximated by (1), the joint torque *τ*_*j*_ can be estimated from (3) by use of (4). However, in actual mechanism, the joint torque could be decreased by mechanical losses such as friction and viscosity of the bag.

## 4. Experiment

**Figure 6:**
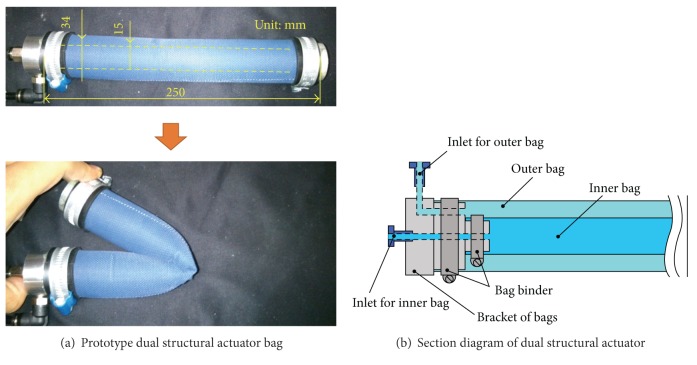
Dual structural actuator.

**Figure 7:**
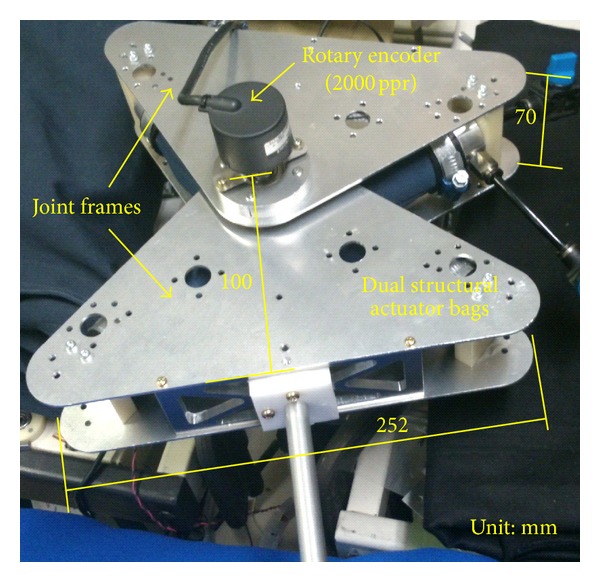
Prototype joint with the actuators.

The prototype actuator is shown in Figure 6. The actuator bag is made of urethane rubber sheets with thermal bonding. It is covered with nylon cloth to enhance the ability to withstand pressure. The nylon cloth is not hermetically sealed; however, it prevents the urethane bag from expanding. With this method, the urethane rubber and nylon cloth are in charge of sealing and structural (mechanical) strength, respectively. This enhancement improves the driving force of the actuator because the allowable input pressure increases. The diameters of the inner and outer actuators are 15 mm and 34 mm, respectively. The effective lengths of each bag are 160 mm (inner) and 200 mm (outer). The withstand pressure of the actuator is about 0.4 MPa.  Figure 6(b) illustrates the section diagram of the actuator. The bracket of the actuator has two fluid paths for the inner and outer actuators.Figure 7 shows the prototype joint with the actuators. The joint frame is made of stainless steel plates and it has 100 mm length, 252 mm width, and 70 mm thickness. The total weight of the joint is 1.3 kg including the actuators. The joint angle is measured every 5 ms by 2000 ppr rotary encoder.

**Figure 8:**
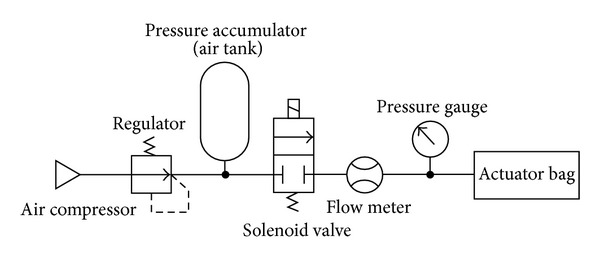
Pressure system for the experiment.

The pressure system of the experiment is illustrated in Figure 8. This experimental system uses air as the driving fluid to reduce the cost of the experiment. The input pressure for the actuator is kept constant by a regulator and accumulator. With this system, the input power can be measured using the flow meter and the pressure gauge. The response times of these meters are less than 5 ms.

**Figure 9:**
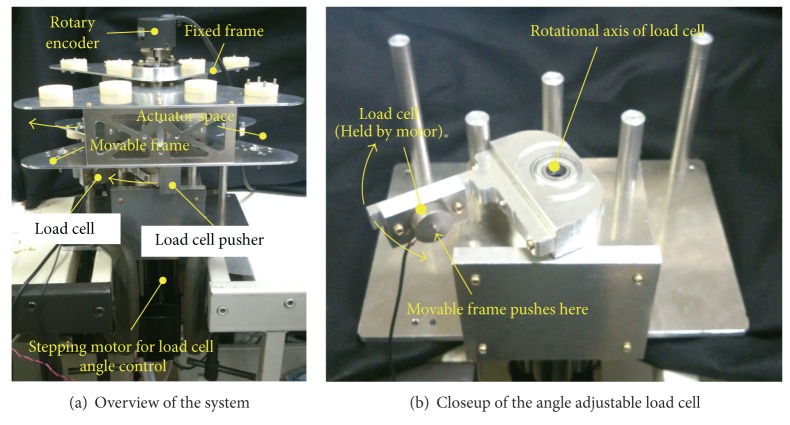
Torque and power measurement system for the prototype joint.

**Figure 10:**
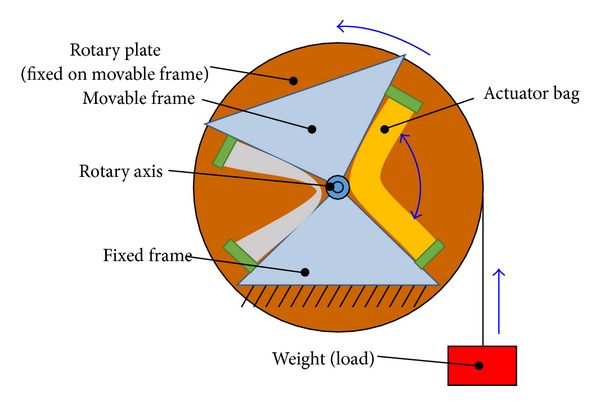
Schematic diagram of load lifting experiment.

Figure 9 shows the torque measurement system with a load cell. The load cell component and one of the joint frames are fixed to the ground. The movable frame of the joint pushes the load cell. The torque is calculated from the force on the load cell. The position of the load cell is controlled by a high torque stepping motor. Since the load cell position is variable, the joint torque can be measured at any angle with high accuracy. This system also can measure the hysteresis of the actuator torque. In addition to this, this study measures work by lifting up a weight using a wire and pulleys.  Figure 10 illustrates the schematic of the load lifting experiment. This system also can measure the instantaneous efficiency by comparing the input and output at any joint angle.

## 5. Results and Discussion

**Figure 11:**
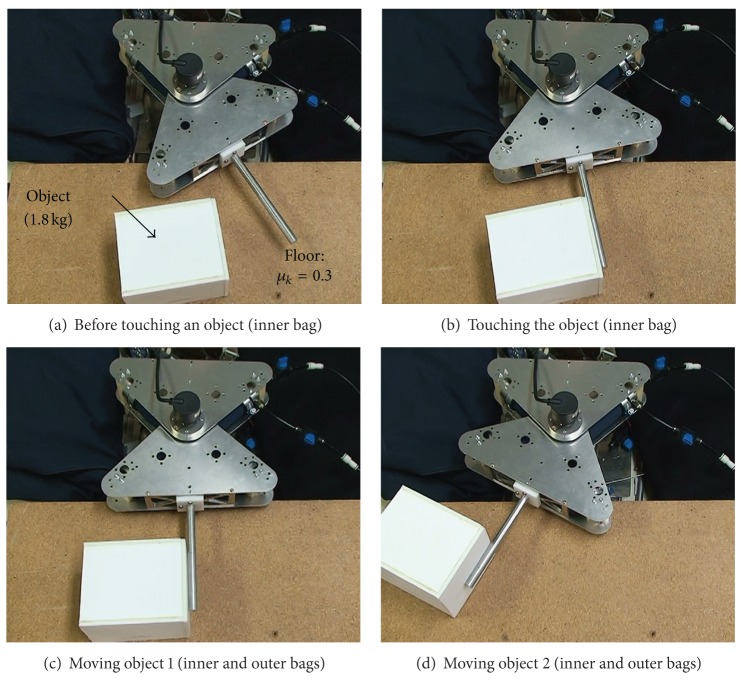
Motion of prototype joint before and after touching an object.

With the proposed mechanism, the inner actuator with narrow width is in charge of position control with a low torque. In contrast, the outer actuator bag is used when a large force is required. The prototype joint is able to control the joint angle by balancing the torques of two opposite actuators. In addition, the joint can change the joint stiffness during the process. The angle and the stiffness are controlled manually at the present stage. For the first step, this study tried a motion with variable output force. The experimental object was a 1.8 kg box on the floor, which has a coefficient of kinetic friction of 0.3 as shown in Figure 11.

Before touching the object, the joint is moved only by the inner bag at a high speed and with a low torque. Such motion mode is appropriate for approaching an object because it saves time and avoids crashing the object (Figure 11(a)). After touching the object (Figure 11(b)), the outer bag starts to generate torque, and the object is moved by the inner and outer bags with a high torque (Figures 11(c) and 11(d)). From this experiment, a coordinated motion of multiple actuator bags for one axis is confirmed with the proposed mechanism.

**Figure 12:**
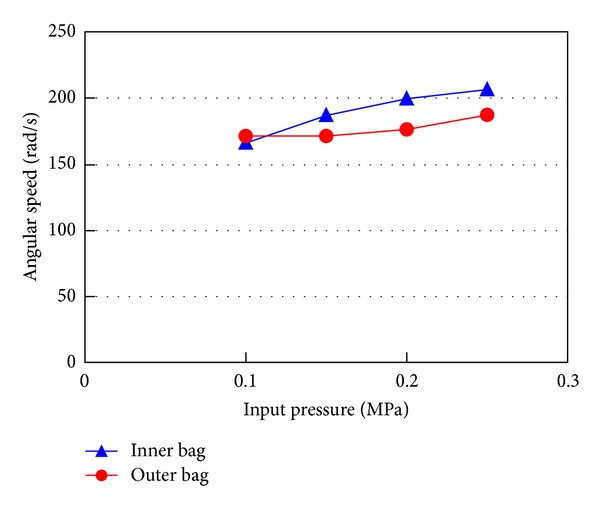
Motion speed comparison between inner and outer bags.

Figure 12 indicates the rotational speeds of the joint moved by inner and outer bags without an external load. The speed of the inner bag is slightly faster than the outer bag. Although the volume of the outer bag is about 6 times larger than the inner bag, the motion speed difference is not very large. A certain motion resistance suppresses the speed of the inner bag because the torque is low. Needless to say, theoretically speaking, the speed of the inner bag is higher than that of the outer bag if the mechanical loss is very small. The use of compressible driving fluid might also explain this result.

However, although the speed difference is not very large, low torque actuator is useful because it avoids crashing an object and saves the amount of driving fluid. If the torque of the inner bag is large enough, for example, when high input pressure is applied or incompressible fluid is used, the required time will be almost proportional to the volume of the bag.

**Figure 13:**
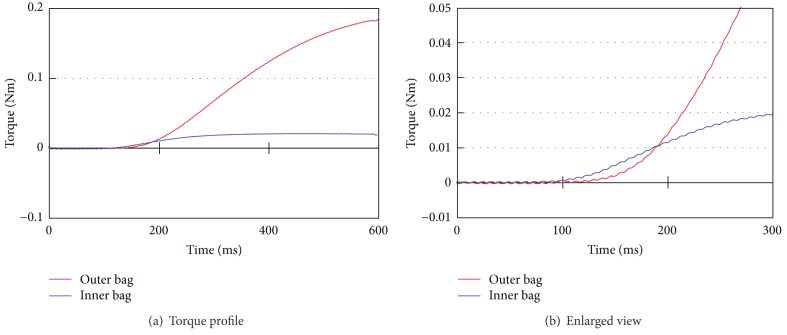
Torque change during pressurization.

Figure 13 shows the joint torque change during actuator pressurization with 0.1 MPa air. Both actuators are depressurized before pressurizing in this measurement. The solenoid valve for the actuator is activated at 0 ms in this graph. From this result (Figure 13(b)), torque increase of the inner bag is a little faster than that of the outer bag around 100 ms. After that, the torque of the inner bag reaches the upper limit near 300 ms. However, the upper limit of the inner bag torque is just only 0.02 Nm, whereas that of the outer bag is about 0.20 Nm near 600 ms (Figure 13(a)).

**Figure 14:**
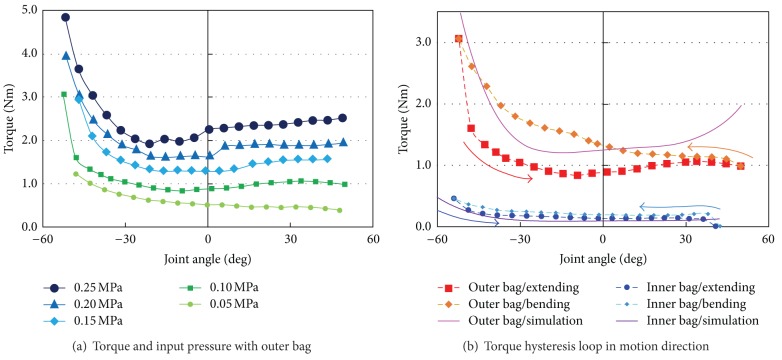
Result of torque measurement.

The result of torque measurement is indicated in Figure 14.  Figure 14(a) represents the outer bag torque dependency on the input pressure. The torque is almost proportional to the input pressure as predicted by (1). Both the inner and outer bags showed the same tendency. The actuator bags also exhibited a hysteresis loop in the direction of motion.  Figure 14(b) shows the hysteresis loop and estimated torque using (1).

In particular, the outer bag shows large loop area. This means that the outer bag is very difficult to bend after expansion under pressurized conditions. This result suggests that a large overshoot should be avoided to control the joint angle when using the outer bag. However, this characteristic is not a serious problem because the outer bag is required large driving force after an object has been grasped. In addition, fast motion of the outer bag is feasible with using a wide pipeline. Meanwhile, the hysteresis loop of the inner bag is very small because the torque is lower than that of the outer bag. This characteristic is appropriate to control the joint angle. Simulation torque curve fits overall results with both actuator bags except for large joint angle areas. This result supports the validity of (1).

The large estimated torque might be caused by the following reason. The reduction ratio of the joint becomes almost infinitely large because the bending angle of the actuator bag is close to zero. It is a singular point. Near this angle range, *dθ*_*j*_/*dθ*_*a*_ in (1) becomes particularly large. However, the actual joint torque is not very large because the actuator bag is not rigid and it has a certain flexibility even if it is pressurized.

**Figure 15:**
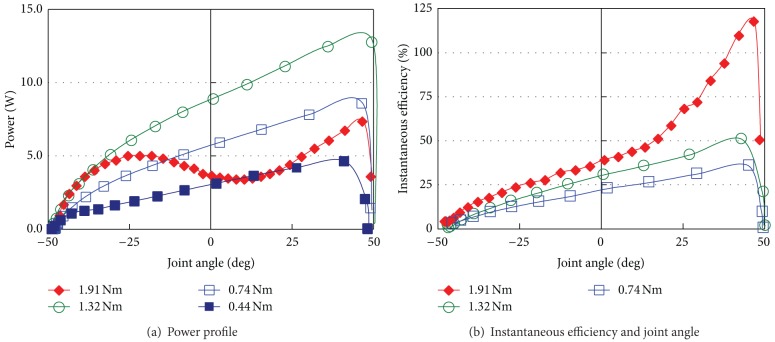
Result of load lifting motion.

With the load-lifting motion, instantaneous power and efficiency are also measured under 0.15 MPa input as indicated in Figure 15.

Under a light load torque, the joint accelerates the speed from the start to the end of the movable angle range. Therefore, the efficiency and power become the maximum near the end of the motion (around large joint angle). In contrast, with a heavy load torque (1.91 Nm), the power of the joint decreases near the large joint angle, whereas the instantaneous efficiency is extremely high. This result is caused by the inertia of the load and the compressibility of air. The result might change if the load is changeable or if the fluid is incompressible.

**Figure 16:**
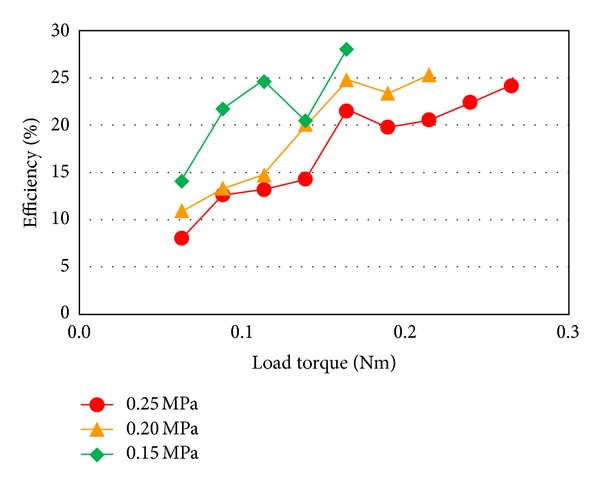
Efficiency dependency on the load torque.

The total efficiency becomes high at high torque range which is observed at power reduction as shown in Figure 15(a). It suggests that the joint mechanism matches the load impedance on this torque range. Since there is a certain motion resistance with the prototype joint, the efficiency decreases when small load torque is applied.  Figure 16 shows the efficiency of the proposed joint. The joint showed about 30% efficiency at 2.0 Nm load torque under 0.15 MPa air input.

## 6. Conclusion

This study newly proposes an active joint with dual structural actuator made of flexible bags. The inner and outer bags are in charge of quick motion and high torque. Coordinated motion of high speed approach and high torque motion are confirmed by the prototype joint. The maximum rotation speed of the joint was about 200 deg/s with the inner bag, while the maximum power of the outer actuator was 13 W under 1.9 Nm load torque. The actuator exhibits a hysteresis loop depending on the direction of motion. The efficiency of prototype joint is about 30%. Feedback control and practical application are our future works.
